# Giant pyoderma gangrenosum in a patient with ulcerative colitis: A case report

**DOI:** 10.1097/MD.0000000000018795

**Published:** 2020-02-07

**Authors:** Ruixian Niu, Jiangtao Zheng, Dongmei Ding, Weian Kuang, Fengyan Lu, Xunguo Yin

**Affiliations:** Department of Dermatology, Qujing First People's Hospital affiliated to Kunming Medical University, Qujing, Yunnan, China.

**Keywords:** pyoderma gangrenosum, ulcerative colitis

## Abstract

**Introduction:**

Pyoderma gangrenosum (PG) is a phenomenon of cutaneous ulceration with unknown etiology. About half the cases have associated extracutaneous manifestations or associated systemic diseases. The most commonly associated systemic disorders include inflammatory bowel disease (IBD), hematologic malignancies, autoimmune arthritis, and vasculitis. This is a case report about giant PG with ulcerative colitis (UC), which is extremely rare.

**Case Presentation:**

A 39-year-old female farmer with UC for the past 3 years presented with multiple painful ulcers, erosion, exudation, and crusting on the right leg for 1 month. A cutaneous examination showed diffusely distributed, multiple, well-defined, deep purulent ulcers on the right medial shank measuring 6 to 20 cm and sporadic worm-eaten ulceration on the right ectocnemial, with severe oozing and erosions. The ulcerations exhibited deep undermined borders, granulated tissue and a black eschar at the base. The right shank and feet were severely swollen, restricting movement. The arteria dorsalis pedis pulse was good, with normal sensation on the skin of the right shank and feet. Laboratory examinations showed a white cell count of 11.8 × 109/L, hemoglobin was 91 g/L, erythrocyte sedimentation rate was 82 mm/h, unelevated procalcitonin, serum C-reactive protein was 131.29 mg/L, and a negative tuberculin skin test. Enteroscopy demonstrated endoscopic evidence of UC. A skin lesion biopsy showed superficial erosion and scarring. Partial epidermal hyperplasia, partial epidermal atrophy and thinning, mild edema of the dermal papill. Most of the middle and lower part of the dermis, showed dense lymphocytes, histiocytes, multinucleated giant cells, and neutrophil infiltration. PG with UC was diagnosed based on clinical manifestations, laboratory examinations and enteroscopy results.

**Interventions:**

She was treated with topical applications of povidone iodine and kangfuxin solution twice daily, methylprednisolone sodium succinate 40 mg and compound glycyrrhizin 60 mg via intravenous drip once a day, along with thalidomide 50 mg twice daily. The UC was controlled with mesalazine.

**Outcomes:**

She required multiple therapies to achieve PG healing 3 months later. No PG recurrence was observed during the 1-year follow-up.

**Conclusion:**

Recognizing the clinical features of PG and its pathogenic nature, ensuring timely management fundamental for preventing severe destruction and deformity, and control of associated diseases are important aspects of treatment. Combination therapy is essential for PG patients with IBD.

## 1 Introduction

Pyoderma gangrenosum (PG) is a rare non-infectious skin disease of undetermined origin. It is characterized by single or multiple painful, necrotic ulcers. Formerly, PG was assumed to be infectious. However, it was established to be an inflammatory disorder. About half of the cases have associated extracutaneous manifestations or associated systemic diseases. The most commonly associated systemic disorders include inflammatory bowel disease (IBD), hematologic malignancies, autoimmune arthritis, and vasculitis. Here, we report a case of giant PG with ulcerative colitis (UC).

## 2 Case report

**Figure 1:**
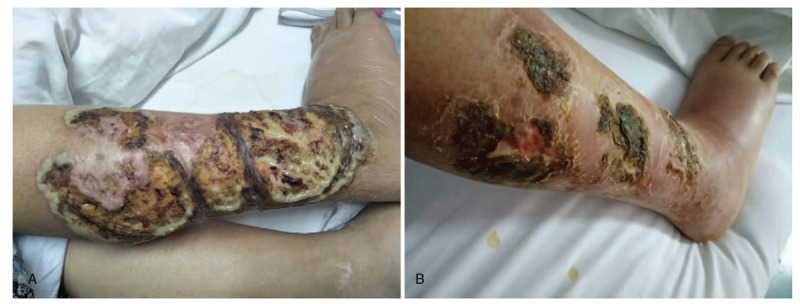
(A and B) Lesions with sporadic worm-eaten ulceration on the right lower limbs.

A 39-year-old woman, who is a farmer presented with multiple painful ulcers, erosion, exudation, and crusting on the right leg for 1 month. One month prior to the current admission, the lesion was erythem, blistered, showing a cricoid or coin shape, with sporadic worm-eaten ulcerations (Fig. 1A and B). She was seen in the orthopedic department, the ulceration was progressively enlarging despite antibiotic treatment, surgical debridement, and negative blood cultures. The severely swollen right shank and feet restricted movement. Bone doctors suggested limb amputation to prevent the skin damage from spreading. However, the patient could not accept the psychological trauma of amputation.

**Figure 2:**
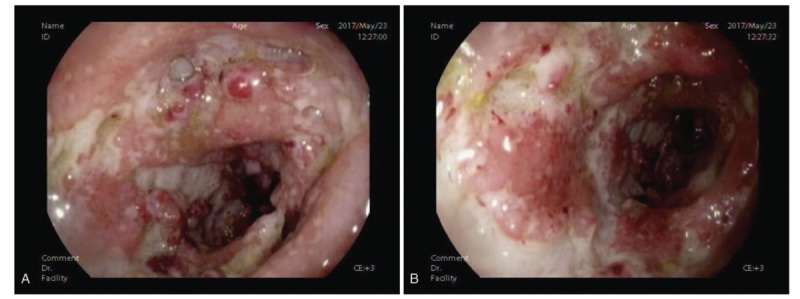
(A and B) Electronic colonoscope examination demonstrating friable deeply ulcerated mucosa in the sigmoid and descending colon.

**Figure 3:**
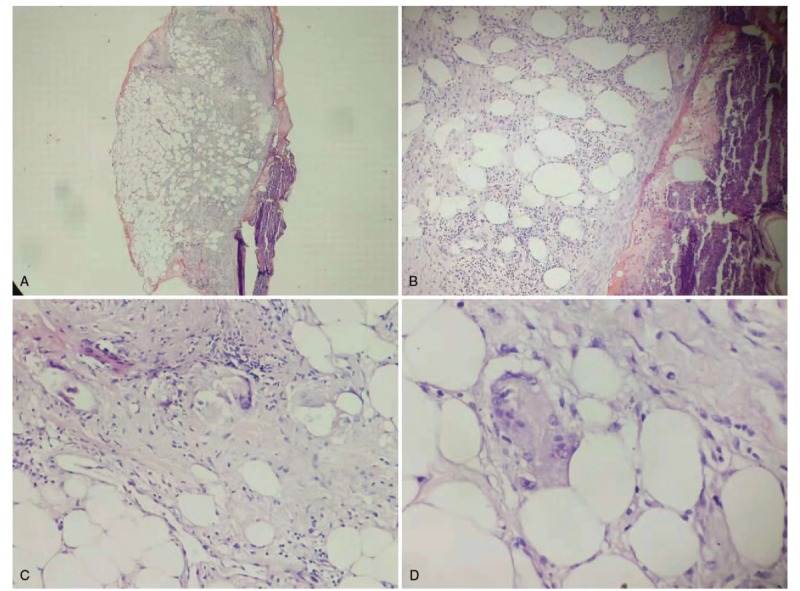
(A) Biopsy examination shows superficial erosion and scarring (H&E×20). (B) Partial epidermal hyperplasia, partial epidermal atrophy and thinning, mild edema of the dermal papilla (H&E×100). (C and D) Most of the middle and lower part of the dermis, shows dense lymphocytes, histiocytes, multinucleated giant cells, and neutrophil infiltration (H&E×200).

The patient came to our in-patient department. Cutaneous examination showed diffusely distributed, multiple, well-defined, deep purulent ulcers on the right medial shank measuring 6 to 20 cm and sporadic worm-eaten ulceration on the right ectocnemial, with severe oozing and erosions. Ulcerations exhibited deep undermined borders and granulated tissue, and a black eschar at the base. The arteria dorsalis pedis had good pulse. The patient had normal sensation on the skin of the right shank and both feet. Oral mucosa, vaginal mucosa, and cutaneous appendages were normal. The systemic examination was unremarkable. Her medical history was significant for intermittent bloody diarrhea for 3 years. Laboratory examinations showed an elevated white cell count of 11.8 × 109/L, 91 g/L hemoglobin, erythrocyte sedimentation rate was 82 mm/h, unelevated procalcitonin, serum C-reactive protein was 131.29 mg/L, and the tuberculin skin test was negative. Enteroscopy demonstrated endoscopic evidence of UC (Fig. 2A and B), hepatic function tests and renal function tests were normal. A skin lesion biopsy showed superficial erosion and scarring. Partial epidermal hyperplasia, partial epidermal atrophy and thinning, mild edema of dermal papill (Fig. 3A). Most of the middle and lower part of the dermis, showed dense lymphocytes, histiocytes, multinucleated giant cells, and neutrophil infiltration (Fig. 3B–D). Based on the clinical manifestation, laboratory examinations and results of the enteroscopy, the diagnosis was giant PG with UC.

**Figure 4:**
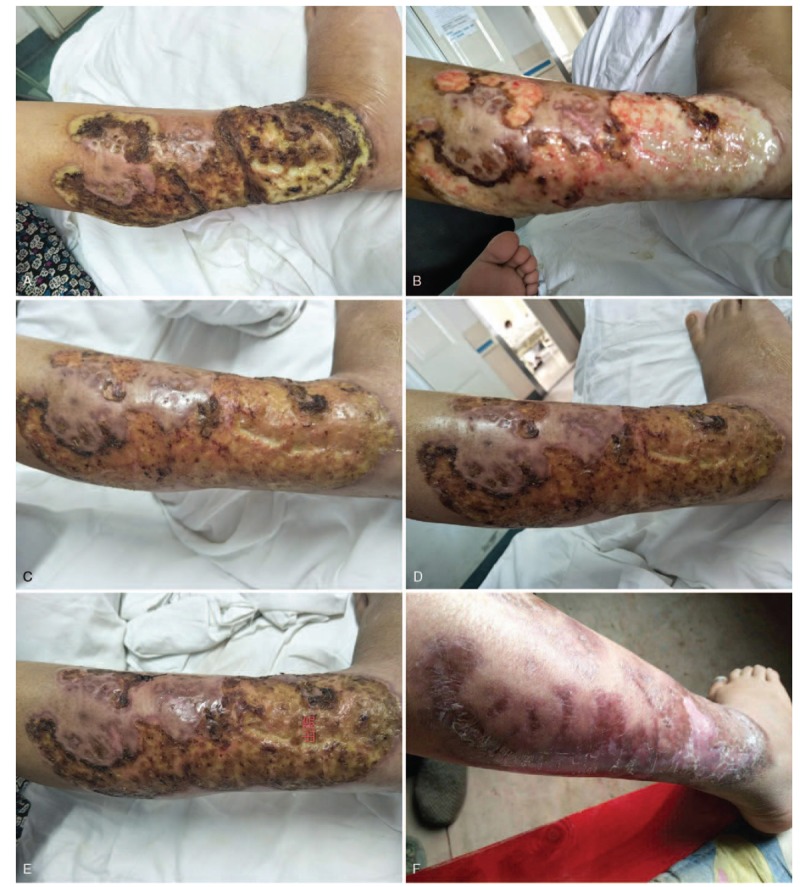
Results of treatment: (A) Within 6 days. (B) At 10 days. (C) At 13 days. (D) At 15 days. (E) At discharge from hospital. (F) After 3 months.

She was treated with topical applications of povidone iodine and kangfuxin solution twice a day, methylprednisolone sodium succinate 40 mg and compound glycyrrhizin 60 mg intravenous drip once a day, along with thalidomide 50 mg twice a day. The UC was controlled with mesalazine. Five days later, the patient showed significant improvement. This patient required multiple therapies to heal the PG after 3 months (Fig. 4A–F).

## 3 Discussion

PG is a cutaneous ulcerative disorder of unknown etiology. Brunsting et al reported the first case in 1930.^[1]^ Due to the rarity of PG, only a few studies in Caucasian-dominant groups have been published, while even fewer reports have addressed Asian populations.^[2]^ As an inflammatory skin disorder, it is usually accompanied by other systemic disorders, including IBD, hematological disorders, systemic lupus erythematosus and rheumatoid diseases.^[2–4]^ PG occurs in 0.5% to 5% of patients with IBD and has previously been shown to be more common in women.^[5]^

Although the exact pathogenesis of PG is unknown, a multifactorial origin including neutrophilic dysfunction, overexpression of mediators of inflammation and genetic mutations predisposing patients to PG have been suggested.^[6]^ Immunological factors and neutrophil dysfunction can be considered to be involved in the etiopathogenesis of PG, such as frequent association of PG with autoimmune diseases and impaired phagocytosis by neutrophils.^[7]^ Since IBD is the most common underlying disorder, cross-reacting antigens in the bowel and skin could be responsible for a secondary cutaneous manifestation.^[8]^ Previous reports suggest that PG and UC, share a common pathogenic immune mechanism, where immune complexes from inflamed intestinal mucosa caused cutaneous lesions, and IL-15 and IL-8 played an important role in the relationship of PG and UC.^[9]^ Minor trauma and surgery are suggested as initiating factors in lesion development.^[6,10]^ Consequently, aggressive surgical wound debridement is contraindicated in these patients.

In the present case, it was delayed diagnosis of PG. The lesions progressively worsened over a 1-week period despite broad-spectrum antibiotics and surgical debridement. Surgical intervention leads to progression of disease. Bone doctors suggested amputation to prevent the skin damage from spreading. The patient could not accept the psychological trauma of amputation. Therefore, prompt and accurate diagnosis of PG is of utmost importance to reduce its often destructive course and psychological pressures.

We reviewed some cases of PG reported in literature and tried to summarize the clinical characteristics, effective management, as well as prognosis. In general, the primary lesions include papules, nodules, pustules, and vesicles, which transform into peripherally growing skin ulcerations.^[6,11,12]^ The morphology of the cutaneous lesions associated with these disorders is heterogeneous and makes the diagnosis challenging. Moreover, a thorough evaluation is required to exclude diseases that mimic these disorders and diagnose potential associated infectious, inflammatory, and neoplastic processes.^[10,13]^ Usually distribution on the lower extremities is most common; however, it has been reported at other sites of the body, such as the nose.^[14,15]^ Overall, 93% of patients with PG had some degree of colonic inflammation.

Underlying bowel disease was active at the time of PG episodes in 65% patients.^[5]^ Other specialists often refer patients with PG to dermatologists after a varying period of time has elapsed without achieving an accurate diagnosis.^[16]^ Currently, the optimal management of PG in this setting is unclear. The variety of treatment strategies used highlights the lack of clear guidelines for treating and managing PG among patients with IBD.^[5]^ Most patients required multiple therapies to achieve PG healing.^[5]^ In literature, multiple treatments, including sunitinib therapy, corticosteroids, cyclosporine, hyperbaric oxygen therapy, and so on with different degrees of improvements.^[3,17,18]^

In the case of our patient, compound glycyrrhizin was one of the treatments used. Glycyrrhizin is the most important and recognized bioactive component of licorice root. The compound is reported to be an effective anti-inflammatory, anticancer, antihepatotoxic, and antioxidant agent, with a glucocorticoid-like effect and was used to manage some diseases, such as systemic lupus erythematous, vasculitis, alopecia areata, and herpes zoster.^[19,20]^ However, as far as we know, it was the first time that compound glycyrrhizin use was reported to treat PG. Moreover, since PG is considered to be associated with some systemic diseases, the management of the disease is also beneficial. As for the current patient, who had significant amelioration without recurrence, the control of UC was quite crucial. In this case, we combined use of corticosteroids, compound glycyrrhizin, thalidomide, mesalazine and added physical therapy. Five days later, a significant improvement was seen in this patient. This patient required multiple therapies to achieve PG healing after 3 months.

In conclusion, we reported a rare case of giant PG in a patient with UC that had good prognosis. It indicated that recognizing the clinical features of PG and its pathogenic nature, while ensuring timely management is fundamental to preventing severe destruction and deformity. Control of associated diseases is also important. Combination therapy is necessary in PG patients with IBD.

## Author contributions

**Resources:** Jiangtao Zheng, Xunguo Yin.

**Software:** weian Kuang.

**Supervision:** Dongmei Ding, Fengyan Lu, Xunguo Yin.

**Writing – review & editing:** Ruixian Niu.
